# Renal denervation as a synergistic tool for the treatment of polymorphic ventricular ectopic beats: A case report

**DOI:** 10.1097/MD.0000000000021098

**Published:** 2020-07-17

**Authors:** Márcio Galindo Kiuchi, Shaojie Chen, Humberto Villacorta, Revathy Carnagarin, Janis M. Nolde, Vance B. Matthews, Markus P. Schlaich

**Affiliations:** a Dobney Hypertension Centre, School of Medicine - Royal Perth Hospital Unit/Medical Research Foundation, University of Western Australia, Crawley, Australia; b Cardioangiologisches Centrum Bethanien (CCB) Frankfurt am Main, Medizinische Klinik III, Agaplesion Markus Krankenhaus, Frankfurt am Main, Germany; c Cardiology Division, Department of Medicine, Universidade Federal Fluminense, Niterói, RJ, Brazil; d Departments of Cardiology and Nephrology, Royal Perth Hospital, Perth, Australia; e Neurovascular Hypertension & Kidney Disease Laboratory, Baker Heart and Diabetes Institute, Melbourne, Australia.

**Keywords:** case report, renal denervation, sympathetic nervous system, ventricular ablation, ventricular arrhythmias

## Abstract

**Introduction:**

Ventricular ectopic beats (VEBs) are very common and often occur in hypertensive or obese individuals, as well as in patients presenting with either sleep apnea or structural cardiac disease. Sympathetic overactivity plays a crucial role in the development, continuation, and exacerbation of ventricular arrhythmias. Recent studies have reported the relevance of sympathetic activation in patients with ventricular arrhythmias and suggested a potential role for catheter-based renal denervation (RDN) in reducing the arrhythmic burden.

**Patient concerns:**

We describe a 38-year-old female symptomatic patient that at the time of presentation was complaining of fatigue in response to minor and medium efforts and not tolerating any physical activity, and episodes of tachycardia associated with dyspnoea, pre-syncope, and syncope.

**Diagnosis:**

She had a high incidence of polymorphic VEBs on 24-hour-Holter monitoring who also presented with left ventricular (LV) hypertrophy for which she was treated with bisoprolol 10 mg/d. The 24-hour-Holter on bisoprolol at baseline showed sinus rhythm with an average heart rate of 92 bpm. There were 44,743 isolated VEBs. A total of 2538 nonsustained ventricular tachycardia events were registered. Her cardiac magnetic resonance imaging showed an increase in LV diastolic diameter and impairment of the right ventricle.

**Interventions:**

The patient underwent endocardial ablation of the right ventricular outflow tract and the LV free lateral wall, and concomitantly underwent bilateral RDN.

**Outcomes:**

Three months post-procedure, her 24-hour-Holter off medication demonstrated an average heart rate 72 bpm and a substantially reduced number of 2823 isolated monomorphic VEBs. Thus far, 18-months follow-up, she has been asymptomatic and doing physical exercises.

**Conclusion:**

In our current patient, we used RDN as a synergistic method to attenuate the sympathetic overactivity, which is narrowly linked to VEBs appearance. Our case report highlighted that RDN may become a potential adjuvant treatment for VEBs in the future.

**Learning points**

- Sympathetic overactivity exerts a crucial role in the development, continuation, and exacerbation of ventricular arrhythmias.
- RDN may become a potential adjuvant treatment for VEBs in the future.

## 1 Introduction

Ventricular ectopic beats (VEBs) are very common, particularly in hypertensive or obese individuals, as well as in patients presenting with either sleep apnea or structural cardiac disease.^[1]^ Overall, VEBs in the structurally normal heart are considered benign.^[2]^ Nonetheless, they have been related to a >2-fold higher risk of cardiovascular events, comprising stroke^[3]^ and cardiac death.^[4]^ Reentry is the probable mechanism for VEBs rising from areas of fibrosis or infiltration in cardiomyopathies, e.g., ischemic cardiac disease, arrhythmogenic right ventricular cardiomyopathy, sarcoidosis, Chagas disease, hypertrophic cardiomyopathies, primary dilated cardiomyopathies, valvular cardiomyopathy, congenital cardiac disease, muscular dystrophies, and metabolic disorders. VEBs can also occur due to reentry around surgical scars. Either small reentry circuits involving the fascicles,^[5,6]^ or triggered or boosted automaticity^[7]^ can provoke fascicular VEBs.

VEBs have recently been identified as a crucial source for deteriorating left ventricular (LV) systolic function, and heart failure in numerous subjects presenting diverse conditions (eg, cardiac ischemia, valvular disease, toxic metabolic or infiltrative conditions, and incessant tachycardia). The pathogenesis of cardiomyopathy mediated by VEBs is unclear, and theories consist of ventricular dyssynchrony, hemodynamic impairment, intensified O_2_ necessity, autonomic dysregulation, modifications in intracellular Ca^2+^ handling, and different heart rate (HR) dynamics.^[8,9]^ Even though VEBs are somewhat infrequent and asymptomatic in most cases, some individuals may sense VEBs more frequently and its symptoms (eg, palpitations, chest pain, dyspnea, and tiredness). The range of benign outflow tract VEBs varies from single VEBs to recurring nonsustained ventricular tachycardia to paroxysmal sustained ventricular tachycardia (VT).^[10]^ Short-coupled right ventricular outflow tract (RVOT) VEBs hardly ever initiate polymorphic VT,^[11]^ whereas even shorter-coupled VEBs frequently coming from the fascicular structure or papillary muscles may trigger ventricular fibrillation.^[7,12]^ Currently, it remains elusive which medical therapy option for treating polymorphic and refractory VEBs may be most beneficial. Possible treatment options involve mapping and ablating them one by one or attempting a novel, alternative, or even adjuvant therapy, aiming to blunt or eliminate different targets.

Sympathetic overactivity plays a crucial role in the development, continuation, and exacerbation of ventricular arrhythmias.^[13]^ Recent studies have reported the relevance of sympathetic activation in patients with ventricular arrhythmias and suggested a potential role for catheter-based renal denervation (RDN) in reducing the arrhythmic burden.^[12]^

## 2 Case presentation

After obtaining written informed consent from the participant for the publication of this case report, we here describe a 38-year-old female patient presenting with a high incidence of polymorphic VEBs on a 24-hour rhythm monitoring (Holter) referred to our Cardiology Service. At the time of presentation, the patient was complaining of fatigue in response to minor and medium efforts and not tolerating any physical activity, and episodes of tachycardia associated with dyspnoea, pre-syncope, and syncope. This was associated with LV hypertrophy on her echocardiogram. She was hospitalized twice due to events of syncope. The episodes started ∼1.5 years ago. She was on bisoprolol 10 mg/d since the beginning of her VEBs. However, her attending physician tried to prescribe metoprolol which she did not tolerate because of her low blood pressure (BP) (111/73 mm Hg, measured in our office) mm Hg, also occurring when she was prescribed sotalol or verapamil. Subsequently, amiodarone was started, but she presented with ophthalmological side effects (perception of colored halos and blurred vision), and the antiarrhythmic medication was discontinued.

Her cardiac magnetic resonance imaging showed an average thickness of the LV inferior-lateral segment, a mild increase in the LV diastolic diameter (5.4 cm, standard range 3.9–5.3 cm), the right ventricle (RV) end-diastolic volume index and the RV end-systolic volume index were reduced (55.1 mL/mm^3^, standard range between 62 and 88 mL/mm^3^; and 33.1 mL/mm^3^, standard range varying between 19 and 30 mL/mm^3^, respectively). Her RV ejection fraction was 40% (normal range: 40%–60%), while her LV ejection fraction was 58.8% (normal range: 50%–70%). No signs of myocarditis, fibrosis, or myocardial necrosis were detected. A 24-hour Holter on bisoprolol 10 mg/d was performed at baseline (Table 1).

**Table 1:**
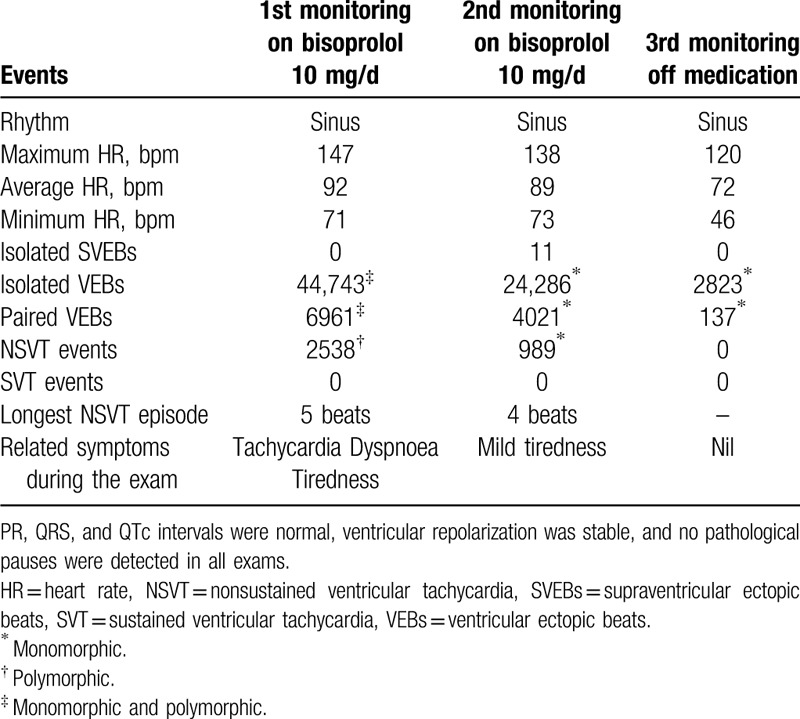
24-hour Holter monitoring.

**Figure 1:**
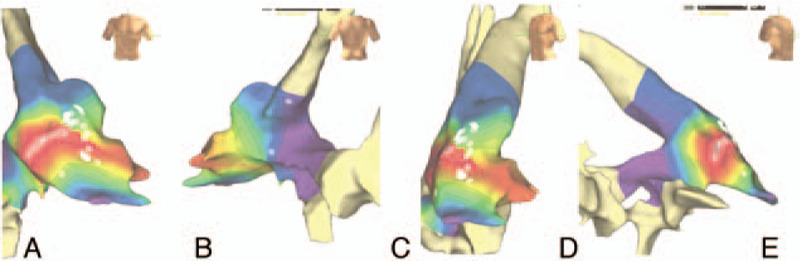
Highest precocity areas (red zones) at the right ventricular outflow tract demonstrated by the electro-anatomical mapping system in 4 different views, anterior (A), posterior (B), lateral-anterior (C), and medial-anterior (D). Thermo-controlled radiofrequency applications (white spots) were performed in the region.

In a joint decision with the patient and her attending physician, we decided to perform a 2-staged catheter ablation procedure. Electrocardiographically, one type of ectopic morphologies, suggested the RVOT as the site of origin. We; therefore, started from the RVOT ectopic beats ablation. In summary, through the Velocity-Ensite 3D mapping system (St. Jude Medical, St. Paul, MN) we detected the highest precocity point at the RVOT (intracavitary electrogram 30 ms earlier than VEBs). We performed thermo-controlled radiofrequency (RF) applications in the region (Fig. 1), and 30 minutes post the last RF application, even in the presence of adrenergic medications, no ectopic activity from the RVOT was observed. No complications occurred, and the patient could be discharged the next day. One month post-procedure, the 24-hour Holter on bisoprolol 10 mg/d was repeated (Table 1).

**Figure 2:**
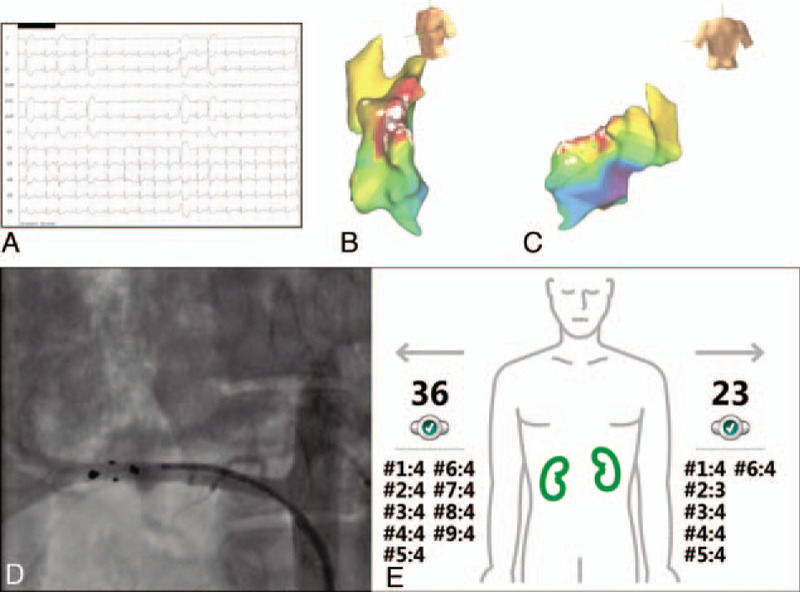
Electrocardiographically, ventricular ectopic beats (VEBs) morphology suggested the free lateral wall of the LV as their site of origin (A). Highest precocity areas (red zones) at the LV free lateral wall demonstrated by the electro-anatomical mapping system in 2 different views, lateral-anterior (B) and posterior (C). The white spots represent thermo-controlled RF applications. The renal denervation catheter was inserted into renal arteries as showed by fluoroscopy (right renal artery) (D), and the EnligHTN system was used to display the number of rounds and ablated spots per renal artery (eg, #1:4 indicates round 1 & 4 ablated spots) (E). 36 = total number of ablated spots in the right renal artery. 23 = total number of ablated spots in the left renal artery. LV = left ventricular, RF = radiofrequency.

After 4 months post-ablation, we scheduled the second procedure. The patient underwent general anesthesia by the anesthesiologist and presented sinus rhythm with frequent VEBs originating from LV. Electrocardiographically, VEBs morphology suggested the free lateral wall of the LV as their site of origin. We performed a transseptal puncture, and positioned the decapolar catheter in the LV, performing the electro-anatomical mapping and reconstruction of this cavity using the aforementioned mapping system. We mapped the LV and found the point of highest precocity on its free lateral wall (Fig. 2). Subsequently, the ablation catheter replaced the mapper catheter into the LV, and RF applications were performed on the free lateral wall of the LV. This resulted in activation of the sites at these moments (NSVT on the ablated sites) and substantially reduced the VEBs from this region. As a final step, we chose to perform percutaneous RDN using the EnligHTN system (St. Jude Medical), aiming to achieve blunting of the sympathetic drive and try to reduce the patient‘s baseline HR and frequency of remaining left VEBs (Fig. 2). Neither cardiac nor vascular complications resulted from the procedures. The patient was discharged 24 hours post-procedures, presenting clinically stable and walking without difficulty.

Three months post-procedure, she underwent another 24-hour Holter off all concurrent medication (Table 1). Also, her mean blood pressure (BP) value taken in our office remained stable at 109/76 mm Hg. Thus far, 18-months follow-up, she has been asymptomatic and doing physical exercises.

## 3 Discussion

Catheter-based RDN is an approach to modulate central afferent input. Renal afferent and efferent nerves work in a reflex loop where afferent input from the kidney to the central nervous system regulates the efferent sympathetic nerve output to the heart and back to the kidney. Both types of renal nerves are mostly situated in the adventitia layer, 1.5 to 2 mm from the lumen of the renal artery.^[14]^ Renal afferent nerves are varied and heterogeneous with myelinated and nonmyelinated fibers.^[15,16]^ Two principal forms of renal afferents relay information to the central nervous system, mechanosensitive (hydrostatic pressure from renal vasculature), and chemosensitive receptors (sensitive to ischemia, osmolar changes, and ionic composition).^[17]^ From a functional perspective, the renal afferent fibers can be categorized into pressor, reno-renal, and depressor types,^[18]^ which means that some of them when activated increase the sympathetic tone, while others depress the sympathetic tone. Preclinical and clinical data support this, pointing out that stimulation of the renal arteries can provoke different responses, including BP elevation.^[19–21]^ Recently, the SPYRAL HTN-OFF MED trial reported that the higher baseline HR was associated with RDN success, which may indicate that RDN works better in patients with a high sympathetic drive (assessed in the absence of interfering drugs),^[22]^ and thereby may also have utility in mitigating arrhythmias through autonomic mechanisms.

Ablation of renal nerves may reduce both systemic and cardiac catecholamine levels, as well as reduce heart fibrosis through modulation of the renin-angiotensin-aldosterone system.^[23,24]^ In a swine model of ischemia, RDN decreased the number of spontaneous VEBs and VF similarly to β-blocker treatment.^[25]^ Also, RDN effects on ventricular electrophysiology seem happen regardless of its effects on BP as shown in initial preclinical and clinical studies.^[26]^ The primary clinical experience with RDN and ventricular arrhythmias has also been encouraging.^[27,28]^ The principal multicenter series of cases, involving 13 subjects with refractory VT, has demonstrated that freedom from VT was assignet to RDN without occurring intraprocedural complications over 3-month follow-up.^[29]^

The gold-standard technique for treating ventricular arrhythmias, which is the endocardial ablation,^[30]^ was employed effectively. However, based on the physiopathology mentioned above, we used additional RDN as a synergic method targeting to attenuate the sympathetic overactivity, which is closely linked to the appearance of VEBs. Our findings, with all limitations of a single case report, suggest that RDN may be a useful adjuvant treatment in this context, a proposition that needs to be adequately tested in the future.

## Author contributions

**Conceptualization:** Marcio Galindo Kiuchi, Shaojie Chen.

**Data curation:** Marcio Galindo Kiuchi, Shaojie Chen.

**Formal analysis:** Marcio Galindo Kiuchi, Humberto Villacorta, Revathy Carnagarin.

**Investigation:** Marcio Galindo Kiuchi, Shaojie Chen, Revathy Carnagarin.

**Methodology:** Marcio Galindo Kiuchi, Shaojie Chen.

**Project administration:** Marcio Galindo Kiuchi.

**Software:** Marcio Galindo Kiuchi, Revathy Carnagarin, Vance Bruce Matthews.

**Supervision:** Humberto Villacorta, Vance Bruce Matthews, Markus Peter Schlaich.

**Validation:** Marcio Galindo Kiuchi, Shaojie Chen, Revathy Carnagarin.

**Visualization:** Humberto Villacorta, Vance Bruce Matthews, Markus Peter Schlaich.

**Writing – original draft:** Marcio Galindo Kiuchi, Humberto Villacorta, Vance Bruce Matthews.

**Writing – review & editing:** Revathy Carnagarin, Markus Peter Schlaich.
